# Advances in Pharmacy Practice: A Look towards the Future

**DOI:** 10.3390/pharmacy10050125

**Published:** 2022-09-30

**Authors:** Jeffrey Atkinson

**Affiliations:** Pharmacolor Consultants Nancy, 12 rue de Versigny, 54600 Villers-lès-Nancy, France; jeffrey.atkinson@orange.fr

**Keywords:** pharmacy, practice, medicine, therapy, industry, future

## Abstract

This review looks at the factors that may influence practice in the future. Transformation could occur at 3 levels. Firstly, the traditional profession of the pharmacist as a dispenser of medicines is expanding. Secondly, the pharmacist’s activities are progressing into new healthcare fields. Thirdly, other changes are stimulated by global developments. This review may be helpful for pharmacy and healthcare leaders looking at the future configuration and aims of their pharmacy services.

## 1. The Emergence of Pharmacy

Throughout the 19th century, pharmacy emerged as an identifiable profession emanating from a nebulous background in which various actors delved in medicinal science and other aspects of healthcare [1,2,3] At the beginning of the 19th century in the United Kingdom, parliament passed an act (1815) stating “*There were four degrees in the medical profession, physicians, surgeons, apothecaries, and chymists and druggists*” [2].

The chemists and druggists developed into pharmacists in the mid-19th century with the foundation of the Royal Pharmaceutical Society in 1841 in the United Kingdom [3]. Similar changes occurred in France in the 19th century following the revolution, with the structuring of the profession and the creation of the first pharmacy faculties leading to a change in training for pharmacists from an apprenticeship to a university degree with examinations, and centralized registration [1].

## 2. The Traditional Role of the Pharmacist as a Dispenser of Medicines

Pharmacists assumed a pivotal role in the treatment stage of the healthcare process with the provision and dispensing of medicines and medical devices, checking of medical prescriptions for safety and interactions, and advising patients (Table 1, stage 3). This role can be divided into substance-oriented and patient-oriented competences Table 2). These competences are associated with legal responsibilities.

At the end of the 20th century, the profession started to evolve further as authorities became aware that the knowledge and skills of the pharmacist could be applied to the prevention of disease, prolongation of life and general improvement of health throughout society [4].

The role of the pharmacist is now evolving in three directions with, firstly, the enlargement of the role of the pharmacist in the area of provision and dispensing of medicines. Secondly, the traditional role in healthcare is expanding as therapeutic tasks that were once the responsibility of the medical practitioner become increasingly shared with pharmacists (and others). Thirdly, changes occur as the pharmacist adapts to climate change.

Several caveats should be born in mind whilst reading this article.

- Current practice differs across countries thus the changes outlined below may already have started in some countries. The review starts from a unifying thread of pharmacy practice in the European Union (EU) then goes onto outline areas in which such practice may develop taking examples from throughout the world.
- Numerous factors drive the changes in the education and practice of pharmacy so the categorization below is explicative, not unmitigated: any given change may arise due to interactions between several causes.
- The effects of some of the changes outlined will not be restricted to pharmacists but will impact other actors in the healthcare professions.

These and other caveats will be discussed at the end of the article.

Pharmacists are medicine specialists in the healthcare process. Medicines are used at all stages of the process: prevention of illness, relief of acute symptoms, treatment of chronic disease, and as antidotes for adverse effects of other medicines. The medicines competencies outlined in Table 1 and Table 2 are derived from the studies of the PHARMINE (Pharmacy Education in Europe) consortium on existing pharmacy education and practice in the EU, and the proposals of the PHARQA (Quality Assurance in European Pharmacy Education and Training) consortium for future education and practice. The work of these two consortia is assembled in the book “Competences for Pharmacy Education and Practice in Europe” [5]. The consortia were composed of academics, practitioners (community, industry, and hospital pharmacists), students, and representatives of EU pharmacy chambers, orders and unions. Periodic reunions to report on progress and obtain feedback were held with the Directorate General for Internal Market, Industry, Entrepreneurship, the executive agency of the European Commission that oversees EU policy on pharmacy education and practice. During this time, the directives issued by this body applied to the 28 member states of the EU, this changed when the UK left the EU in January 2020. 

## 3. Evolution of the Traditional Role of the Pharmacist in the Provision and Dispensing of Medicines

### 3.1. Changes in Medicines—Low M.Wt. Chemicals to High M.Wt. Biologics

There is an ongoing change from therapy with low M.Wt. chemical substances to that with high M.Wt. biologics [6,7]. The latter are often nucleic acids, antibodies, enzymes, and other types of protein. Initially, this will affect specialized, hospital pharmacy then fundamental, community practice. 

In the late 18th and early 19th centuries the discovery of endogenous chemical transmitters, and their receptors, gave rise to the receptor theory for the development of molecules mimicking neurotransmitters and hormones. From the 1950s onwards, the medicines developed were primarily small M.Wt. molecules such as aspirin and paracetamol. These were researched by pharmacological receptor theory and then developed and produced by chemical methods. These new drugs appeared in a ready-to-use form reducing the need for the preparation of individual prescriptions. They were largely orally administered. 

Nowadays, large M.Wt. biomolecules or biologics are starting to replace these medicines [6]. Although biologics are researched using receptor theory as before, they are developed by biomolecular sciences, immunology, and genetic engineering, and produced with biotechnological processes. Biologics harbor the promise of better targeted therapy for diseases therebefore lacking in suitable remedies (certain viral diseases, vascular diseases such as macular degeneration in the eye, certain forms of cancer, etc.). They present new formulation challenges with innovative delivery systems, and novel pharmacokinetics of the active principle.

The expansion of the use of biologics is impacted by the economics and politics of therapy. Given that the pharmaceutical/biotechnological companies producing medicines work within a market economy, this expansion is affected by economic factors. The most profitable medicines, often the most expensive, now coming onto the market are of biomolecular/biotechnological origin [8]. Some are used in the top therapeutic classes such as anti-cancer treatment [9]. Thus they may be prescribed to substantial numbers of patients. The authorization for use of a medicine entails a preliminary discussion on the equilibrium between recuperation by the company of the (large) amount of money invested in research and development, and the amount the government can afford to pay for treatment. An example of this was seen in the discussions over the purchase price of the new mRNA vaccines for COVID-19 and the implications of increased transparency in financial transactions on the availability of vaccines in economically less well-developed countries [10]. 

In the immediate future, most prescription drugs will remain (relatively) cheap, small M.Wt., chemical molecules. Between 2006 and 2009 British academics and National Health Service specialists developed a core list of the one hundred most prescribed medicines. This was revised in 2018. The list was stable over the decade concerned. The top three drug classes (estrogens and progestogens, phosphodiesterase inhibitors and serotonin receptor agonists) were small M.Wt. substances [11].

In the future pharmacists will have to adapt to the use of large M.Wt. biologics with complex pharmacotherapy, unique formulation and pharmacokinetics of substances often administered parenterally. As many are heat-labile they may require specific transport and storage facilities. Pharmacists may also have to adapt to a changing economic situation in which therapies are more expensive. 

The development of biosimilars (follow-on or subsequent entry biologics) may attenuate the increase in the price of treatment [12]. Pharmacists can play a key role in the introduction and use of biosimilars [12]. Okoro [12] has proposed a specialty of “biotherapeutic pharmacy”. Educational interventions by specialists in this subject could overcome the fears and misconceptions over the use of biosimilars (and biotherapeutics in general), advise on the addition of biosimilars to the healthcare formulary, organize the storage and dispensing of biosimilars, and play an essential role in clinical trials of biologics. Albeit a relatively recent systematic review of physicians’ perceptions on the uptake of biosimilars showed that most (64–95%) showed concerns over pharmacist-led substitution of biologic medicines [13]. This goes to show once again that although a certain development in pharmacy practice may have a solid, logical starting point in terms of the competencies of the pharmacist, progress in that area will also depend on acceptance by the triad: policymaker, patient and healthcare player.

### 3.2. Pharmacogenomics

A major change in medicinal therapy is the development of pharmacogenomics. Pharmacogenomics is the study of how an individual’s genetic make-up can affect a person’s response to medicines. The use of genetic testing and pharmacogenomics optimizes medicine selection and dosage. It holds the promise of individually tailored therapy providing greater efficacy with fewer side effects [14]. It also may allow for the reintegration of medicines with actionable pharmacogenomic data. Classic pharmacogenomic markers were associated with metabolizing enzymes. Potential exists for the use of such data in the treatment with oncology-related medicines [15] and also in psychiatry [16], infectious [17], and cardiovascular [18] diseases. 

For this specialized practice, pharmacists will need the required competencies. Pharmacogenomics can provide valuable pharmacodynamic and pharmacokinetic information that can be used by pharmacists in the assessment and choice of drug therapy [19]. Pharmacists‘ roles may include the promotion of the optimal use and timing of pharmacogenomic tests, interpretation of the results of such tests, and education of healthcare professionals, patients and the public about the field of pharmacogenomics [20].

### 3.3. IT (Informational Technology) and AI (Artificial Intelligence)

Advances in IT are having a major impact on practice [21]. One example is the development of electronic prescriptions [21]. This ensures greater reliability and safety in repeated prescriptions in chronic illness and ameliorates the sharing of dispensing information between community and hospital pharmacists and between pharmacists and medical doctors. 

Progress in IT combined with AI is opening new areas in tele-medicine and tele-dispensing. Tele-medicine is not only driven by such developments but also by decisions on policy and healthcare governance based on the relative responsibilities and cost effectiveness of different professionals [9]. 

IT and AI have been used in the creation of treatment algorithms for the monitoring of chronic treatment. For instance, pharmacist-managed warfarin protocols with computerized systems are being used to calculate the anticoagulant dose based on algorithms incorporating clinical factors and the International Normalized Ratio (INR) [22]. This system improves INR control with less bleeding in higher risk surgical patients. The authors suggested that “*centralisation of inpatient management within specialty pharmacy-led practice could enable improvements in safety and quality metrics related to all anticoagulants, not just warfarin*.” 

Pharmacists have key leadership roles in healthcare driving the implementation, use and development of IT/AI services for electronic prescribing, dispensing, and prescription transfer.

### 3.4. Prescribing

#### 3.4.1. Rectification of Prescriptions and Care Transfer

Approximately one in thirty patients are subject to medication harm that could be avoided [23] and in 25% of cases such harm is life-threatening. Pharmacist screening together with other methods could help in the detection of this problem. In a hospital pharmacy, a frequent clinical incident is medication-related error [24]. These errors often occur during admission and discharge of patients, raising questions about the interaction of the hospital pharmacist with other hospital staff, and with the patient’s community pharmacist. In the Beks et al. study in seven Australian hospitals from 2016 to 2017, a “Partnered Pharmacist Medication Charting” model was established in which participating pharmacists completed a credentialing program to equip them with the skills needed to participate in medication-charting. The study showed that special training of hospital pharmacists in medication charting with other healthcare professionals can improve the situation [24].

This raises a general problem of IT/AI systems for transfer of care systems (for review see [25]). Several countries are developing pharmacist-led electronic care transfer systems to decrease the risks attached to discharge from hospital such as medication errors, care discontinuity, etc. with possible necessity for readmission. Several issues are at stake such as feedback, practitioner accountability, and automated flexibility for referrals. This move towards an IT/AI solution could be determinant in freeing healthcare staff capacity in a cost-effective manner and, finally, improving patient safety.

#### 3.4.2. Dispensing without Prescription and Prescription by Pharmacists

The pharmacist is legally responsible for the act of dispensing only. In some situations, however, responsibility goes beyond that, and pharmacists can lawfully dispense medicines without prescription. One example is the dispensing of opioid antagonists such as naloxone to rapidly reverse opioid overdose [26,27]. 

Pharmacists can also prescribe medicines in certain situations. Pharmacist prescribing is one of the most fundamental changes to the role of the profession and many countries are now rolling out pharmacist prescribing services. The latter requires prior post-registration experience of several years and completion of a curriculum on non-medical prescribing [28]. One example is pharmacist prescription of emergency contraception with hormones such as levorgestrel [29]. There is some evidence that pharmacists can prescribe to the same standards as doctors and that they may make significantly fewer prescribing errors when charting patients’ medicines upon admission to the hospital [30]. Further studies are required on the impact of pharmacist prescribing on patient safety. 

#### 3.4.3. Antimicrobial Resistance

Antimicrobial resistance is brought about by inappropriate prescribing (especially in primary care) and sales, use of antibiotics outside the healthcare system, bacterial genetic changes, and insufficient investment in the development of new antibiotics [31]. Antimicrobial resistance is a major problem in primary care where viruses cause most infections. Here, pharmacists can play a decisive role in programs to promote antimicrobial stewardship [32,33]. 

Antimicrobial stewardship can include optimizing therapy by recommending an appropriate medicines regimen, duration of therapy and dosage route (switch from intravenous to oral), monitoring of therapy, and instructing healthcare workers and patients on the appropriate use of antibiotics. Pharmacists can also play a role in the use of antibiotics outside the healthcare system for instance in the case of unlawful dispensing of prescription-only medicines. This can be problematic with medicines such as antibiotics where such practice can increase the risk of developing antibiotic resistance [34]. 

## 4. Other Roles of Pharmacists in Healthcare

### 4.1. Public Health

Their 5–6-year degree course gives pharmacists in-depth knowledge of disease and therapy. In the context of ever-increasing healthcare budgets, governing agencies are looking at the cost-efficiency of the different actors in the global healthcare scheme and the ways to put pharmacy competencies to better economic use. Thus, the role of pharmacists in governmental policies are changing with greater involvement in the general health of the population [35,36]. Examples of this are the roles pharmacists play in smoking cessation [37], limitation of excessive alcohol consumption and weight management [38], and syringe and needle exchange [39]. 

The evolution of the role of community pharmacists from substance- or product-oriented services to patient-centered services has given rise to the creation of the discipline of pharmaceutical care. This involves the management of acute situations such as chlamydia testing [40] and emergency contraception [29], and chronic diseases such as diabetes [41], arterial hypertension [42], HIV/AIDS, [43], hyperlipidemia [44], asthma [45], kidney disease [46], urinary tract infection [47], and depression [48]. There is also potential for pharmacist expertise in palliative care [49] and organ transplantation [50]. 

The effectiveness of pharmacists was confirmed in a meta-analysis of reviews published from 2007 to 2017 on the impact of community pharmacist-led interventions in chronic disease management [51]. Intervention improved adherence to medication, reduced admission rates for heart failure, and improved lung function in patients with respiratory conditions. The impact of pharmacists in primary care has been studied in another meta-analysis of thirty-eight reports [35]. The surveys recruited patients with cardiovascular disease or diabetes. Pharmacist interventions involved medication review delivered collaboratively with the general practitioner. These interventions lead to significant improvements in blood pressure, glycosylated hemoglobin and cholesterol. 

Competencies for pharmacy practice will further develop within the context of an interactive, global healthcare complex of responsibilities. One aspect is the creation of healthcare centers grouping the activities of pharmacists and other professionals (doctors, nurses…) [9,52]. This allows a more in-depth examination and management of therapy with a reduction in the multiplication of visits to different healthcare specialists often separately located. 

### 4.2. Vaccination

A recent example of the involvement of pharmacists in primary care is given by vaccination campaigns. Pharmacists are increasingly integrated into vaccination campaigns, not only in community pharmacies alongside general medical practitioners, but also in hospitals and vaccination centers [53,54]. Pharmacist-led influenza vaccination campaigns are now commonly used to reach inoculation targets and increase vaccination coverage. In 2012–2013, the EU coverage for influenza was only 53% for over 65s [55]. In areas where community pharmacists were commissioned to vaccinate, rates in over sixty-fives were much higher [56]. 

The role of pharmacists in fighting infectious diseases was highlighted by the recent COVID-19 pandemic. The importance of pharmacists is underlined by the rapid response measures needed to mitigate the spread and impact of COVID-19 [54]. This latter European study showed that the pharmacists’ role involves both their substance-oriented and patient-oriented competencies: organization of the supply and storage of (sometimes heat-labile) vaccines, distribution and delivery of vaccines, reconstitution of vaccine preparations, patient education, as well as actual vaccination. 

The impact of the COVID-19 pandemic on practice is judged by many to be a major milestone in the recent history of pharmacy. Bragazzi, et al. [57] judge that “*The COVID-19 outbreak has unearthed new opportunities for pharmacists: community and hospital pharmacists have, indeed, played a key role during the COVID-19 pandemic, suggesting that a fully integrated, inter-sectoral and inter-professional collaboration is necessary to face crises and public health emergencies*” and go on to conclude that “*a new era in the history of pharmacies (‘the post-COVID-19 post-pharmaceutical care era’) has begun, with community pharmacists acquiring a more professional standing…”*. The impact of the COVID-19 pandemic is such that many countries have changed or are envisaging changing the legislation on the responsibilities of pharmacists. Thus, in an Australian study Lee, et al. [58] concluded that “*Modifying legislation to allow pharmacists to administer approved COVID-19 vaccines will enable a trained and skilled workforce to be deployed to increase the rate of mass vaccination”*.

### 4.3. Aging

The aging of the population, in many (but not all) countries, challenges the satisfactory treatment of the chronic (often neuronal and cardiovascular) diseases that afflict senior citizens. This involves changes in the classes of medicines prescribed and the ways in which they are dispensed within the context of the increasing complexity of multiple co-morbidities leading to polypharmacy [9]. Co-morbidity is one of the causes of inappropriate polypharmacy (excessive use of unnecessary medicines), especially in the elderly suffering from multimorbidity [59]. In the UK more than half of those over 65 are prescribed ›3 medicines, the most common being antihypertensives, lipid-lowering medicines, proton-pump inhibitors, analgesics and antidepressants [60]. A recent report by the Chief Pharmaceutical Officer of the UK [61] stressed the importance of the pharmacist in providing structured medication reviews as the solution to this problem. Distinguishing appropriate from inappropriate polypharmacy by limiting overprescribing is a complex subject involving different combinations of medications with different risk/benefit ratios, administered to patients with different clinical backgrounds. On-going, pharmacist-led IT/AI projects could help with this complex, multifactorial problem [59]. 

Polypharmacy for the multimorbidity of the elderly is increasing due to several factors such as rising levels of multimorbidity with the aging of the population, disease-specific rather than patient-specific prescribing guidelines, and lack of evidence to support deprescribing [62,63]. This has several potentially negative effects: reduced adherence, adverse effects of medicines, increased utilization of healthcare services, frailty, cognitive impairment, and mortality. Most chronic diseases of the elderly are treated with medicines, thus pharmacists, with their extensive knowledge of pharmacotherapy and pharmacokinetics, can play a unique role in optimizing pharmacotherapy and decreasing polypharmacy [64]. To date reports on the impact of pharmacists are mixed [64] and more studies are needed to evaluate the real impact of the pharmacist and the ways in which this could be amplified. 

One other potential role for the pharmacist with the aging population concerns the possible increase in allo- and xenotransplantation following end organ damage. Despite the protective effects of chronic pharmacotherapy on prevention of organ failure produced by chronic diseases such as arterial hypertension, diabetes, vascular disease, etc. aging is associated with progressive decline in function of the heart, kidneys, and other organs. This will potentially increase the demand for transplantation. There is evidence that transplantation pharmacists can improve outcomes such as adherence, morbidity and medication errors connected to transplantation [50].

The problems arising from the increase in longevity will also be met by enhanced use of IT and AI for home support and telemedicine [65], and biological revolutions such as the application of pharmacogenomics to diagnosis and choice of therapy. The final result will be a new form of “5P” care: predictive, preventive, personalized, participative, and proven. Pharmacists will have a primordial role to play in such progress [9].

## 5. Climate Change and the Pharmacist

There are two aspects of climate change for pharmacists: how can pharmacists fight climate change and how can they fight the healthcare effects of climate change [66]. Examples of the first are the production of greenhouse gases by pharmaceuticals in manufacturing, transportation, and disposal through incineration, the hydroxy-fluoroalkane propellants used in metered-dose inhalers, and the contamination of water systems through effluent from the manufacturing of medicines, and human excretion [67]. 

An example of the second impact is given by zoonotic pandemics caused by viruses provoking HIV/AIDS, severe respiratory syndromes, and influenza, which originate in animals displaced by ecological, behavioral and/or socio-economic changes [68]. Climate change and the subsequent destruction of the habitats of mammals will modify the development and distribution of zoonoses. Modeling suggests that in the future, climate change will drive mammal migration to cooler regions providing new opportunities for virus exchange. Virus sharing will occur mainly in densely populated areas such as India and Indonesia [69]. Veterinary pharmacists with their unique knowledge of human and non-human physiology, pathology and therapy, are ideal partners for teams involved in prediction, prevention, and therapeutics of zoonoses. 

Climate change and the ensuing migrations may also produce changes in the ethnic distribution of populations and hence in the pharmacogenomic responses to medication. Here, it should be noted that ethnic minorities are underrepresented in the clinical trials on future medicines and efforts to solve this clearly identified problem have proven elusive [70]. Again, pharmacists with, for example, their knowledge of medicinal science and especially pharmacogenomics will have a significant role to play in selecting those patients that may benefit from treatment with medicines that are not necessarily adequately adapted to their ethnic group as a whole.

## 6. Caveats and Conclusions

This review describes the factors determining the future development of pharmacy and the responsibilities of the pharmacist. Wide-ranging changes are occurring for instance in the biotechnological revolution in medicines, the switch to more patient-based practice, dispensing, and zoonoses provoked by climate change.

In conclusion, several observations are opportune.

- This review is not intended as an all-inconclusive, in-depth analysis but a wide-ranging survey. The author accepts that this will lead to frustration for some readers faced with what they may consider as inadequate, shallow treatment of a particular subject. The review includes many references that can be used to enlarge knowledge in each area.
- The initial chapters on the framework and status of current practice are based on the author’s experience in Europe (for key articles see [5]). Most European countries have a federated legal healthcare system with exceptions such as Switzerland that has a combination of national and subnational (cantonal) levels [71]. The legal framework covers the workforce and operations (premises, processes, etc.)*,* license and ownership, and service and its remuneration. Concerning the latter many countries are developing and/or have developed provisions to improve access to medicines and pharmaceutical expertise such as extending dispensing, online medicine sales, etc. (see above for examples from the United Kingdom). In addition to the above national instruments, the twenty-seven member states of the EU (with an overall population of 450 million people) are under the obligation to apply the directive of the Directorate General for Internal Market, Industry, Entrepreneurship [72] that defines the framework of (mainly community) pharmacy education and service throughout the EU. It should be noted that this directive (latest version: EU, 2013) is issued by the Internal Market directorate –not the health directorate—of the EU. Its prime aim is to ensure free movement of healthcare professionals throughout the EU independently of the member state in which they received their education and training. At a national level there is a wide range of education and practices from more “substance-based” (e.g., Denmark) to more “patient-oriented” (e.g., France and Estonia). The EU 2013 directive does not recognize pharmacy specialties (such as industrial and hospital pharmacy) which are recognized (especially for hospital pharmacy) at a national level, often in the form of several years of internship following the pharmacy first degree, in many EU countries. The role of the industrial pharmacist is identifiable in the EU directive and national legislations on qualified persons [73,74,75]. In the light of the above, differences in the current practice across countries in the EU mean that future development will differ as countries have different starting points. This is also true for countries elsewhere throughout the world in some of which pharmacists have been present in hospital wards for decades and have an established role in minor ailments in the community pharmacy.
- Issues of society, economics and policy will affect many if not all the changes outlined above. For instance, in both publicly and privately funded healthcare systems, the relative cost efficiencies of the different actors (medical doctors, pharmacists, nurses, et al.) will have to be considered, and the pharmacist may have certain advantages [76]. In this context, advantages of the pharmacist include:
  o Pharmacists receive an extensive and in-depth training in pharmacodynamics and pharmacokinetics
  o There are organizational advantages to the involvement of pharmacists in primary care. Access to pharmaceutical services is often available without an appointment. Pharmacies are often open for longer periods during the day and at weekends. They are often situated in deprived areas with few primary care providers. They treat elderly patients with comorbidities on poly-medication that see their pharmacists on a regular basis. A recent American study [77] showed that the ratio of pharmacist to physician encounters was 13:8 in metropolitan areas and even higher at 14:5 in rural areas. A study in England showed that almost 90% of the population have access to a community pharmacy within a 20-min walk. In areas of highest deprivation almost 100% of the population have access within a 20-min walk. The authors proposed the concept of “a positive pharmacy care law” viz higher access to a pharmacy in areas of highest deprivation [78].
- There is a transfer of pharmacists’ traditional activities to others. For example, a recent change in laws on practice was seen during the COVID-19 pandemic: the possibility for supermarkets and other retail outlets to sell medical devices such as face masks. For the time being, such changes are minor.

In conclusion, this review briefly describes the traditional profession of the pharmacist as a dispenser of medicines and how this role is expanding. It goes on to discuss how the activities of the pharmacist are progressing into new healthcare fields. Finally, the effect on pharmacy of global climate change is described. This review may be helpful for pharmacy and healthcare leaders looking at the future configuration and aims of their pharmacy services, and at policies on how to reply to future changes.

## Figures and Tables

**Table 1:** The traditional healthcare process.

Stage	Stage 1: Assessment	Stage 2: Initiation of Treatment	Stage 3: Treatment
Actions	Health and risk assessment	Diagnosis, prescription, and/or modification of treatment	Treatment: preventive or curative
Responsibility	*Medical practitioner* Clinical examination Clinical biology analyses Medical history Screening for disease Lifestyle		*Pharmacist* Provision and dispensing of medicines Checking of medical prescriptions for safety and interactions Advice to patients *Medical practitioner* Prescription of medicines Surgery Vaccination Psychiatry *Mixed* Lifestyle advice

**Table 2:** The traditional pharmacist’s competencies as a provider and dispenser of medicines.

Substance-Oriented Competencies	Patient-Oriented Competencies
Research and development of active medicines Dose and formulation of medicines Pre-clinical and clinical evaluation of fitness for purpose of medicines (efficacy, safety) Production, distribution, storage, and marketing of medicines Economics and policy on the use of medicines	Dispensing of medicines Diagnostic procedures, clinical biology guiding the use of medicines Patient counselling on medicines Patient counselling on lifestyle and other factors affecting the prescription and efficacy of medicines Pharmacovigilance of primary and secondary actions of medicines, and their interactions

## Data Availability

Not applicable.
